# Apixaban, an orally available anticoagulant, inhibits SARS-CoV-2 replication and its major protease in a non-competitive way

**DOI:** 10.1093/jmcb/mjac039

**Published:** 2022-06-27

**Authors:** Otávio Augusto Chaves, Carolina Q Sacramento, Natalia Fintelman-Rodrigues, Jairo Ramos Temerozo, Filipe Pereira-Dutra, Daniella M Mizurini, Robson Q Monteiro, Leonardo Vazquez, Patricia T Bozza, Hugo Caire Castro-Faria-Neto, Thiago Moreno L Souza

**Affiliations:** Laboratory of Immunopharmacology, Oswaldo Cruz Institute (IOC), Oswaldo Cruz Foundation (Fiocruz), Rio de Janeiro, RJ, 21040-900, Brazil; National Institute for Science and Technology on Innovation on Neglected Diseases (INCT/IDN), Center for Technological Development in Health (CDTS), Oswaldo Cruz Foundation (Fiocruz), Rio de Janeiro, RJ, 21040-900, Brazil; Laboratory of Immunopharmacology, Oswaldo Cruz Institute (IOC), Oswaldo Cruz Foundation (Fiocruz), Rio de Janeiro, RJ, 21040-900, Brazil; National Institute for Science and Technology on Innovation on Neglected Diseases (INCT/IDN), Center for Technological Development in Health (CDTS), Oswaldo Cruz Foundation (Fiocruz), Rio de Janeiro, RJ, 21040-900, Brazil; Laboratory of Immunopharmacology, Oswaldo Cruz Institute (IOC), Oswaldo Cruz Foundation (Fiocruz), Rio de Janeiro, RJ, 21040-900, Brazil; National Institute for Science and Technology on Innovation on Neglected Diseases (INCT/IDN), Center for Technological Development in Health (CDTS), Oswaldo Cruz Foundation (Fiocruz), Rio de Janeiro, RJ, 21040-900, Brazil; National Institute for Science and Technology on Neuroimmunomodulation (INCT/NIM), Oswaldo Cruz Institute (IOC), Oswaldo Cruz Foundation (Fiocruz), Rio de Janeiro, RJ, 21040-900, Brazil; Laboratory on Thymus Research, Oswaldo Cruz Institute (IOC), Oswaldo Cruz Foundation (Fiocruz), Rio de Janeiro, RJ, 21040-900, Brazil; Laboratory of Immunopharmacology, Oswaldo Cruz Institute (IOC), Oswaldo Cruz Foundation (Fiocruz), Rio de Janeiro, RJ, 21040-900, Brazil; Institute of Medical Biochemistry Leopoldo de Meis, Federal University of Rio de Janeiro (UFRJ), Rio de Janeiro, RJ, 21941-590, Brazil; Institute of Medical Biochemistry Leopoldo de Meis, Federal University of Rio de Janeiro (UFRJ), Rio de Janeiro, RJ, 21941-590, Brazil; Laboratory of Immunopharmacology, Oswaldo Cruz Institute (IOC), Oswaldo Cruz Foundation (Fiocruz), Rio de Janeiro, RJ, 21040-900, Brazil; National Institute for Science and Technology on Innovation on Neglected Diseases (INCT/IDN), Center for Technological Development in Health (CDTS), Oswaldo Cruz Foundation (Fiocruz), Rio de Janeiro, RJ, 21040-900, Brazil; Laboratory of Immunopharmacology, Oswaldo Cruz Institute (IOC), Oswaldo Cruz Foundation (Fiocruz), Rio de Janeiro, RJ, 21040-900, Brazil; Laboratory of Immunopharmacology, Oswaldo Cruz Institute (IOC), Oswaldo Cruz Foundation (Fiocruz), Rio de Janeiro, RJ, 21040-900, Brazil; Laboratory of Immunopharmacology, Oswaldo Cruz Institute (IOC), Oswaldo Cruz Foundation (Fiocruz), Rio de Janeiro, RJ, 21040-900, Brazil; National Institute for Science and Technology on Innovation on Neglected Diseases (INCT/IDN), Center for Technological Development in Health (CDTS), Oswaldo Cruz Foundation (Fiocruz), Rio de Janeiro, RJ, 21040-900, Brazil

## Dear Editor,

The severe coronavirus disease 2019 (COVID-19) is associated with coagulopathy. Anticoagulants, such as low-molecular-weight heparin, warfarin, thrombin inhibitors, and factor Xa (FXa) inhibitors, are thus recommended by the American Society of Hematology and National Institutes of Health for COVID-19 patients (Wenzler et al., 2020; Adam et al., 2021). Clinical trials with anticoagulants have shown the increased survival of critically ill COVID-19 patients under non-invasive and invasive ventilatory assistance (Wenzler et al., 2020; Adam et al., 2021), along with decreased consumption of platelets and clotting factors and a reduced risk of hemorrhage (Adam et al., 2021). Among the anti-clotting agents, early use of orally available FXa and thrombin inhibitors (Chowdhury et al., 2020; Rentsch et al., 2021) prevented high levels of D-dimer, which is the final product from the clotting/fibrinolysis cascade and is directly implicated with severe COVID-19 (Rentsch et al., 2021).

Curiously, the active binding pockets of FXa, thrombin, and the severe acute respiratory syndrome coronavirus 2 (SARS-CoV-2) main protease (M^pro^) share a considerable similarity, as judged by the superimposition of their 3D structures (Supplementary Figure S1; Biembengut and De Souza, 2020). Although structural similarities between FXa and thrombin with M^pro^ have been suggested, functional studies to indicate whether M^pro^ could use FXa or thrombin inhibitors or substrates are scarce. Thus, we interrogated whether (i) FXa inhibitors (apixaban and rivaroxaban) and a thrombin antagonist (dabigatran) could inhibit M^pro^ activity and SARS-CoV-2 replication and (ii) M^pro^ could directly cleave FXa substrate.

**Figure 1:**
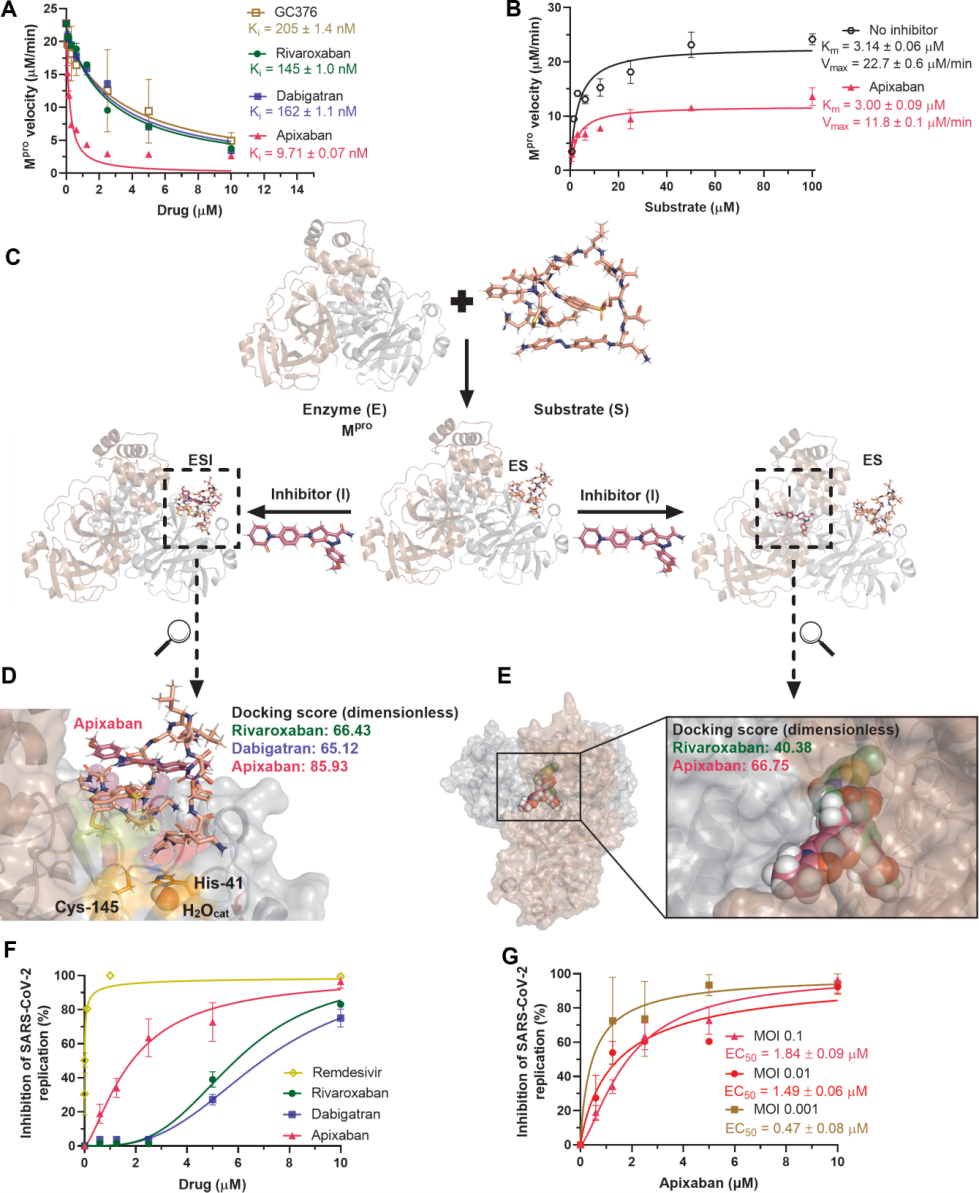
Apixaban inhibits SARS-CoV-2 replication and M^pro^ activity in a non-competitive way. (**A**) The enzymatic inhibition profile for apixaban, rivaroxaban, dabigatran, and GC376 (positive control) for 88.8 nM M^pro^, assayed by commercial FRET kit (BPS Biosciences Ltd, #79955-1). (**B**) Michaelis–Menten enzymatic inhibition for M^pro^ in the absence or presence of a fixed apixaban concentration (2.5 µM) for different substrate concentrations. (**C**) Enzymatic scheme for the mechanism of M^pro^ inhibition by apixaban. (**D**) Best docking pose (ChemPLP function) for the interaction between the M^pro^/peptide (ES) complex and apixaban (ESI) into the active site of the protease. (**E**) Best docking pose (ChemPLP function) for the interaction between the dimer interface of M^pro^ with apixaban and rivaroxaban. Substrate, rivaroxaban, and apixaban are in stick representation in beige, green, and pink, respectively, while the catalytic water (H_2_O_cat_) is in a sphere. The monomers that constitute the active M^pro^ structure are in cartoon representation in brown and gray. Elements’ colors: hydrogen, nitrogen, oxygen, sulfur, and chloro are in white, dark blue, red, yellow, and dark green, respectively. (**F**) Antiviral activity of anticoagulants and remdesivir (positive control) in Calu-3 cells (2.0 × 10^5^ cells/well) infected with SARS-CoV-2 under a MOI of 0.1. (**G**) Apixaban activity in terms of percentage of viral replication in Calu-3 cells (2.0 × 10^5^ cells/well) under three different MOIs.

Among the anticoagulants tested, apixaban was the most potent to inhibit M^pro^ activity, with Morrison's inhibitory constant (*K*_i_) value of 9.71 nM (Figure 1A; see also Supplementary material for methodological details). Apixaban was 21-fold more potent than GC376, used here as a positive control. Of note, the FXa inhibitor rivaroxaban and the thrombin inhibitor dabigatran were as potent as GC376 (Figure 1A). Curiously, apixaban inhibits M^pro^ activity with a *K*_i_ lower than the concentration of the viral protease used in the assay, and thus a non-canonical mechanism of inhibition over this enzyme could be expected. When apixaban's inhibition over M^pro^ was assayed under different concentrations of the substrate, a non-competitive mechanism was observed (Figure 1B). The Michaelis–Menten constant (*K*_m_) value was not altered by apixaban, indicating that M^pro^ was not halted to interact with its substrate by this drug (Figure 1B). In addition, there was a significant decrease in the M^pro^ maximum velocity (*V*_max_) by apixaban (Figure 1B), indicating that M^pro^ was unable to cleave its substrate adequately in the presence of apixaban. Based on our data, the enzyme (E) M^pro^ may interact with its substrate (S) and form an ES complex even in the presence of apixaban, but subsequent cleavage of the peptidic substrate is impaired by this drug (as exemplified in Figure 1C).

We envision two possible ways by which non-competitive inhibitors affect enzymatic activity: (i) the ES complex conformation creates new opportunities for drugs to inhibit product formation; and/or (ii) the inhibitor interacts with allosteric sites important for enzyme catalysis. Both hypotheses were evaluated by *in silico* calculations.

On the M^pro^/peptide (ES) complex, the peptidic substrate used in the enzymatic assays interacts with the enzyme by occupying four subsites (S1, S1′, S2, and S4) in the active site, with a docking score (dimensionless) of 65.54 (Supplementary Figure S2A). In this ES complex, the substrate forms an external loop, which may be targeted by the anticoagulants (Supplementary Figure S2B). In particular, apixaban had the most favorable interaction, compared with dabigatran and rivaroxaban, due to the hydrogen bonding interaction with the substrate (3.00 Å) (Figure 1D; Supplementary Figure S2C and D). Based on molecular docking, it is feasible that apixaban targets the substrate in the ES complex.

Moreover, to test the possibility that apixaban could target an allosteric site, we followed the molecular docking calculations from El-Baba et al. (2020), which identified allosteric regions on the M^pro^ dimeric interface. We found that apixaban could potentially interact with an allosteric site on M^pro^, supported by a high number of hydrogen bonding and van der Waals interactions (Figure 1E; Supplementary Figure S2E). For comparison, rivaroxaban did not reach the allosteric site; the

M^pro^/M^pro^ dimeric interface alone and dabigatran did not show a feasible binding capacity into these additional sites (Figure 1E; Supplementary Figure S2F).

Interestingly, Papaj et al. (2022) reported a weak interacting capacity of apixaban with M^pro^ without the presence of a peptidic substrate. This is in line with our data because both enzymatic assays and *in silico* calculations indicated that apixaban is a non-competitive inhibitor, meaning that its effect on M^pro^ is facilitated when the ES complex is formed. Based on Papaj et al. (2022), we also interpret that apixaban on the allosteric site could be weak; otherwise, these authors would have found apixaban direct binding on M^pro^ by thermal shift assay. Using this biophysical readout, Papaj et al. (2022) only observed apixaban's effect at the concentration ≥10 µM under M^pro^ concentration of 4 µM. To measure enzymatic activity, we used a lower enzyme concentration, 88 nM, which favors observing apixaban's potency at lower concentrations. A combination of biochemical and biophysical assays, e.g. thermal shift, surface plasmon resonance, and, most importantly, structural experiments, should be performed to further clarify how apixaban could target M^pro^.

To interpret the biological significance of M^pro^ inhibition by the anticoagulants, we performed SARS-CoV-2 infection in Calu-3 cells followed by treatment with these drugs. Calu-3 recapitulates the most affected cells in the respiratory tract, type II pneumocytes (Chu et al., 2020). Anticoagulants indeed inhibited the production of infectious SARS-CoV-2 progeny in a dose-dependent manner (Figure 1F; Supplementary Table S1). Consistently with the enzymatic data, apixaban was ∼3-fold more potent than the other tested anti-clotting

drugs. Nevertheless, apixaban was ∼60-fold less potent *in vitro* than the positive control, remdesivir (Figure 1F; Supplementary Table S1). Additionally, despite the slightly higher cytotoxicity compared to other tested compounds, apixaban was 2-fold better in selectivity index (SI) for SARS-CoV-2 replication than the other tested anticoagulants (Supplementary Table S1).

During the clinical evolution of COVID-19 patients, FXa antagonists may be used as early or late intervention (Wenzler et al., 2020; Adam et al., 2021; Rentsch et al., 2021). Viral loads are usually high after the onset of illness and tend to decrease even for patients who progress to poor clinical outcomes. Thus, we tested whether the potency of apixaban to inhibit SARS-CoV-2 replication in Calu-3 cells could be altered as a function of the virus input. Indeed, we observed that apixaban displayed a multiplicity of infection (MOI)-dependent activity (Figure 1G). Our results show a consistent pattern of apixaban's effect on M^pro^ activity and the inhibition of viral replication, which reinforces the necessity of further structural studies to precisely evaluate how apixaban's chemical structure is associated with M^pro^ complexed with its substrate to even allow further hit-to-lead development of specific antivirals against COVID-19.

Although anticoagulants inhibit SARS-CoV-2 replication by targeting M^pro^, the ability of viral proteases (both M^pro^ and papain-like protease) to use FXa (S-2765) and thrombin (S-2238) substrates was absent (Supplementary Figure S3). In fact, host and viral enzymes belong to different families of endopeptidases, and M^pro^ lacks the superimposed random coils that are external to the active site of FXa, which might impact the mimetic chromogenic substrate accommodation into the M^pro^ active site (Supplementary Figure S3).

Under clinically approved posology of 10 mg, apixaban reaches a maximum plasmatic concentration (*C*_max_) of 0.55 mM (Byon et al., 2019). Considering that 87% of apixaban is bound to albumin (He et al., 2011), its free fraction at *C*_max_ is equivalent to 72 nM, almost 10 times higher than apixaban's *K*_i_ toward M^pro^. Apixaban's potency against SARS-CoV-2 *in vitro* replication was MOI-dependent, ranging from lower to three times higher than human *C*_max_. The viral load is lower at the late stages of COVID-19 when apixaban and other anti-clotting agents were originally proposed and could contribute to accelerating the decline in viral RNA levels. At the early stages of diseases, when viral loads are higher, it is more likely that the main mechanism of action over FXa is more pronounced than any effect on the inhibition of virus replication.

It is naturally difficult to estimate the clinical benefit of any antiviral activity of apixaban during clinical trials because its anti-clotting activity is directly associated with COVID-19 physiopathology. Our results indicate that apixaban, besides its anti-clotting activity, may inhibit SARS-CoV-2 replication and M^pro^ activity. We consider the apixaban chemical structure as a lead to be optimized for the development of novel non-competitive M^pro^ inhibitors that preserve anticoagulant activity.

*[This work was supported by the Brazilian agencies Conselho Nacional de Desenvolvimento CientÍfico e Tecnológico (CNPq, 441019/2020-0 and 307162/2017-6) and Fundação de Amparo à Pesquisa do Estado do Rio de Janeiro (FAPERJ, E-26/210.182/2020, E-26/201.067/2021, and E-26/210.112/2020). This study was financed in part by Coordenação de Aperfeiçoamento de Pessoal de Nível Superior (CAPES, Brazil, 88887.506989/2020-00). Funding was also provided by CNPq, CAPES, and FAPERJ through the National Institutes of Science and Technology Program on Diseases of Neglected Populations (INCT-IDPN, 465313/2014-0). O.A.C. thanks Dumith Chequer Bou-Habib and Fundação para o Desenvolvimento Científico e Tecnológico em Saúde (FIOTEC) both from Oswaldo Cruz Foundation for the grant VPPCB-005-FIO-20. The featured image was created with BioRender.com. T.M.L.S., H.C.C.-F.-N., and P.T.B. idealized the work. O.A.C., C.Q.S., N.F.-R., and J.R.T. conducted the SARS-CoV-2 inhibition in Calu-3 cells. O.A.C., F.P.-D., and L.V. conducted the experimental enzymatic assays. O.A.C. conducted the cytotoxic assays and molecular docking calculations. D.M.M. and R.Q.M. conducted the pro-clotting coagulation assays. O.A.C., R.Q.M., H.C.C.-F.-N., and T.M.L.S. prepared the letter to the editor.]*

## Supplementary Material

mjac039_Supplemental_File
