# Supplementary material for: Apixaban, an orally available anticoagulant, inhibits SARS-CoV-2 replication and its major protease in a non-competitive way
Source: J Mol Cell Biol. 2022 Jun 27;14(6):mjac039. doi: 10.1093/jmcb/mjac039 (PMC9492308; doi:10.1093/jmcb/mjac039)
Supplement: mjac039_Supplemental_File [file mjac039_supplemental_file.pdf]

Supplementary material

Apixaban, an orally available anticoagulant, inhibits SARS-CoV-2 replication and its major protease in a non-competitive way

Otávio Augusto Chaves[1,2,*], Carolina Q. Sacramento[1,2], Natalia Fintelman-Rodrigues[1,2], Jairo Ramos Temerozo[3,4], Filipe Pereira-Dutra[1], Daniella M. Mizurini[5], Robson Q. Monteiro[5], Leonardo Vazquez[1,2], Patricia T. Bozza[1], Hugo Caire Castro-Faria-Neto[1], and Thiago Moreno L. Souza[1,2,*]

[1]Laboratory of Immunopharmacology, Oswaldo Cruz Institute (IOC), Oswaldo Cruz Foundation (Fiocruz), Rio de Janeiro, RJ, Brazil

[2]National Institute for Science and Technology on Innovation on Neglected Diseases (INCT/IDN), Center for Technological Development in Health (CDTS), Oswaldo Cruz Foundation (Fiocruz), Rio de Janeiro, RJ, Brazil

[3]National Institute for Science and Technology on Neuroimmunomodulation (INCT/NIM), Oswaldo Cruz Institute (IOC), Oswaldo Cruz Foundation (Fiocruz), Rio de Janeiro, RJ, Brazil

[4]Laboratory on Thymus Research, Oswaldo Cruz Institute (IOC), Oswaldo Cruz Foundation (Fiocruz), Rio de Janeiro, RJ, Brazil

[5]Institute of Medical Biochemistry Leopoldo de Meis, Federal University of Rio de Janeiro (UFRJ), Rio de Janeiro, RJ, Brazil

*Correspondence to: Otávio Augusto Chaves, E-mail: otavioaugustochaves@gmail.com; Thiago Moreno L. Souza, E-mail: tmoreno@cdts.fiocruz.br

Supplementary Material and methods

General materials

All chemical-reagents were purchased from Sigma-Aldrich/Merck (St. Louis, MO, USA), HyClone Laboratories Inc. (Logan, Utah) or Chromogenix (Diapharma Group, Inc., KY).

Cells and virus

Human lung epithelial (Calu-3) and African green monkey kidney (Vero, subtype e6) cells were cultured in high-glucose Dulbecco’s modified eagle medium (DMEM—HyClone, Logan, UT, USA) supplemented with 100 U/mL penicillin, 100 µg/mL streptomycin (P/S—Thermo Fisher

Scientific®, Waltham, MA, USA) and 10% fetal bovine serum (FBS—HyClone, Logan, UT, USA). The cells were incubated at 37 ℃ in 5% of carbon dioxide (CO_2). The SARS-CoV-2 B.1 lineage (GenBank #MT710714) was isolated on Vero E6 cells from nasopharyngeal swabs of confirmed cases. All procedures related to virus culture were handled at biosafety level 3 (BSL3) multiuser facility at *Fundação Oswaldo Cruz* (FIOCRUZ), Rio de Janeiro, Brazil, according to World Health Organization (WHO) guidelines (Bain et al., 2020).

Enzymatic assays

The anticoagulants capacity in inhibit enzymatic velocity of main protease (M^{pro}) of SARS-CoV-2 was determined by the commercial kit provided by BPS Bioscienсses® company (catalog number: #79955-1) following the procedure and recommendations from literature and manufacturer (di Sarno et al., 2021; Glaab et al., 2021). This enzymatic kit is based on FRET assay using a substrate peptide of M^{pro} labeled with a fluorescent dye (Dabcyl) and an acceptor−quencher (Edans) at the *N*- and *C*-terminus, respectively. The substrate peptide does not fluoresce in the uncleaved state, where the quencher blocks the fluorescence of the dye. However, after M^{pro} cleaves the substrate, the fluorescence of the dye is dequenched and an emission signal is observed. The inhibitor blocking the activity of M^{pro} prevents the FRET-peptide cleavage and a low fluorescence signal will be observed. Basically, 88.8 nM M^{pro} was incubated overnight in reaction buffer (20 mM Tris pH 7.3, 100 mM NaCl, 1 mM EDTA, 1 mM DTT, and 1 μM BSA) containing 25 μM of substrate (modified peptide DABCYL-KTSAVLQSGFRKME-EDANS, with CAS number 730985-86-1) and each anticoagulant or GC376 as positive control (Ma et al., 2020; Fu et al., 2020) at concentrations of 0.0, 0.08, 0.16, 0.31, 0.63, 1.25, 2.5, 5.0, and 10 μM. Fluorescence signal was measured at 460 nm upon excitation at 360 nm in a GloMax® (Promega, Madison, WI, USA) plate reader. The Morrison's inhibitory constant (K_i) value was calculated by non-linear regression using GraphPad Prism 9. The Michaelis–Menten plot was conducted for 88.8 nM M^{pro} incubated overnight in assay buffer with substrate concentrations varying from 0 to 100 μM in the presence and absence of 2.5 μM of apixaban. After fluorescence quantification, the Michaelis–Menten constant (K_m) and maximum velocity (V_{max}) were also calculated by non-linear regression using GraphPad Prism 9 (Windows GraphPad Software, San Diego, CA, USA). For all the cases, the values were presented as mean ± standard deviation (SD).

Cleavage of chromogenic substrate assays

The M^{pro} and papain-like protease (PL^{pro}) were assayed for amidolytic activity towards the hydrolysis of the synthetic chromogenic substrates for thrombin (S-2238) and coagulation factor Xa (S-2765). Hydrolysis of S-2238 and S-2765 (0.2 mM final concentration) by M^{pro} (10 nM) and

PL^{pro} (10 nM) was determined in 50 mM Tris/HCl, 150 mM NaCl, 10 mM $CaCl_2$, and 0.1% polyethylene glycol (PEG) 6000, pH 7.5. Substrate hydrolysis was detected using a SpectraMax® ABS Plus equipped with a microplate mixer and heating system. Reactions were recorded continuously at 405 nm for 2 h at 37 ℃.

Yield-reduction assays and virus titration

The Calu-3 cells were infected with multiplicity of infection (MOI) of 0.1 at densities of 2.0×10^5 cells/well for 1 h at 37 ℃ in 5 % of CO_2. The cells were washed, and different concentrations of each anticoagulant or remdesivir were added in Dulbecco's modified Eagle medium (DMEM) with 10% fetal bovine serum (FBS). The concentration of anticoagulants was 0.00, 0.63, 1.25, 2.50, 5.00, and 10.0 µM, while remdesivir was 0.00, 0.0001, 0.001, 0.01, 0.10, 0.50, 1.0, 5.0, and 10.0 µM. After 48 h, the supernatants were harvested, and virus titers were quantified by plaque-based assays according to previous publications (Fintelman-Rodrigues et al., 2020; Puhl et al., 2021; Sacramento et al., 2021).

To perform the virus titration, Vero cells (2.0×10^4 cell/well) in 96-well plates (Nalge Nunc Int, Rochester, NY, USA) were infected with log-based dilutions of the yield reduction assays' supernatants for 1 h at 37 ℃ in 5% of CO_2. After the incubation, medium containing 1.8% CMC with 5% FBS was added and incubated at 37 ℃ with 5% CO_2 for 72 h. The cells were fixed with 10% formaldehyde in PBS and stained with a 0.04% solution of crystal violet in 70% methanol. The virus titers were calculated by scoring the plaque-forming unit (PFU/mL) and a non-linear regression analysis of the dose–response curves was also performed to calculate the 50% effective concentration (EC_{50}). All experiments were carried out at least three independent times, including a minimum of two technical replicates in each assay, and each data was analyzed from GraphPad Prism 9.0 (Windows GraphPad Software, San Diego, CA, USA). The value was presented as mean ± standard deviation (SD).

Cytotoxic assays

The cytotoxic assays were conducted in a monolayers of Vero cells (in about 2.0×10^4 cell/well) treated for 3 days with different concentrations of apixaban, rivaroxaban, dabigatran, or remdesivir (50, 150, 300, 600, and 800 µM) following procedure described in literature (Sacramento et al. 2021). The plates were read in terms of absorption in a spectrophotometer at 595 nm and the 50% cytotoxic concentration (CC_{50}) was calculated by a non-linear regression analysis from a dose–response curve.

Molecular docking procedure

The crystallographic structure for M^{pro} was obtained from Protein Data Bank, with access code 7K40. The chemical structure for the anticoagulants and M^{pro} substrate used in the experimental assays was built and minimized in terms of energy by Density Functional Theory (DFT) via Spartan'18 software (Wavefunction, Inc., Irvine, CA, USA). The molecular docking calculations were performed with GOLD 2020.2 software (Cambridge Crystallographic Data Center Software Ltd., CCDC) at pH 7.4. Redocking studies were carried out with the crystallographic ligand boceprevir (GC376), obtaining the lowest RMSD value by ChemPLP function. The main binding pockets for drugs into M^{pro} were calculated by the free access software from Proteins Plus (Zentrum für Bioinformatik, Universität Hamburg, Germany). It was defined 8 Å radius around each main binding pockets and the figures of the best results were generated with PyMOL Delano Scientific LLC software (DeLano Scientific LLC: San Carlos, CA, USA).

Supplementary References

Bain, W., Lee, J.S., Watson, A.M. et al. (2020). Practical guidelines for collection, manipulation and inactivation of SARSCoV-2 and COVID-19 clinical specimens. Curr. Protoc. Cytom. *93*, e77.

di Sarno, V., Lauro, G., Musella, S. et al. (2021). Identification of a dual acting SARS-CoV-2 proteases inhibitor through in silico design and step-by-step biological characterization. Europ. J. Med. Chem. *226*, 113863.

Fintelman-Rodrigues, N., Sacramento, C.Q., Lima, C.R., et al. (2020). Atazanavir, alone or in combination with ritonavir, inhibits SARS-CoV-2 replication and proinflammatory cytokine production. Antimicrob. Agents Chemother. *64*, e00825.

Fu, L., Ye, F., Feng, Y. et al. (2020). Both Boceprevir and GC376 efficaciously inhibit SARS-CoV-2 by targeting its main protease. Nat. Comm. *11*, 4417.

Glaab, E., Manoharan, G.B., Abankwa, D. (2021). Pharmacophore model for SARS-CoV‐2 3CLpro small-molecule inhibitors and in vitro experimental validation of computationally screened inhibitors. J. Chem. Inf. Model *61*, 4082-4096.

Ma, C., Sacco, M.D., Hurst, B. et al. (2020). Boceprevir, GC-376, and calpain inhibitors II, XII inhibit SARS-CoV-2 viral replication by targeting the viral main protease. Cell Res. *30*, 678-692.

Puhl, A.C., Fritch, E.J., Lane, T.R., et al. (2021). Repurposing the ebola and marburg virus inhibitors Tilorone, quinacrine, and pyronaridine: In vitro activity against SARS-CoV-2 and potential mechanisms. ACS Omega *6*, 7454–7468.

Sacramento, C.Q., Fintelman-Rodrigues, N., Temerozo, J.R., et al. (2021). In vitro antiviral activity of the anti-HCV drugs daclatasvir and sofosbuvir against SARS-CoV-2, the aetiological agent of COVID-19. J. Antimicrob. Chemother. *76*, 1874–1885.

Supplementary Figures

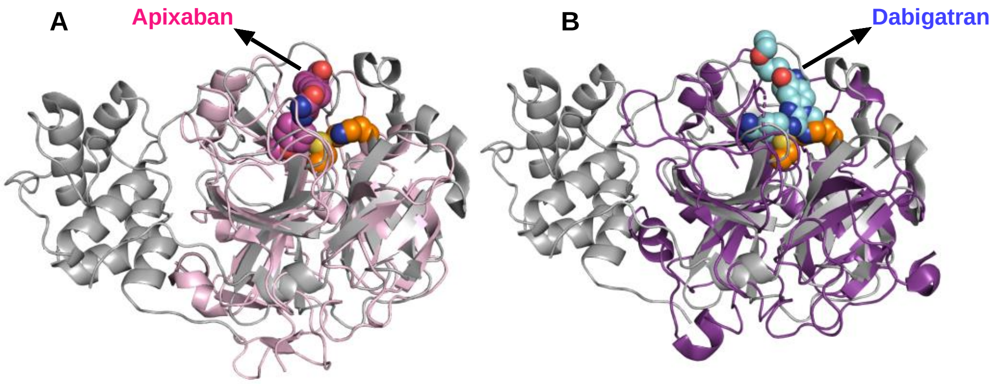

Supplementary Figure S1. Superposition of the monomeric unit of M^{pro} (in gray, PDB code 7K40) with (**A**) FXa (in violet, PDB code 2P16) and (**B**) thrombin (in purple, PDB code 1KTS). The crystallographic structure of apixaban into FXa structure, dabigatran into thrombin structure, and catalytic dyad of M^{pro} (His-41 and Cys-145 residues) are as spheres in pink, cyan, and orange, respectively. For better interpretation the catalytic water (H_2O_{cat}) of M^{pro} was not shown.

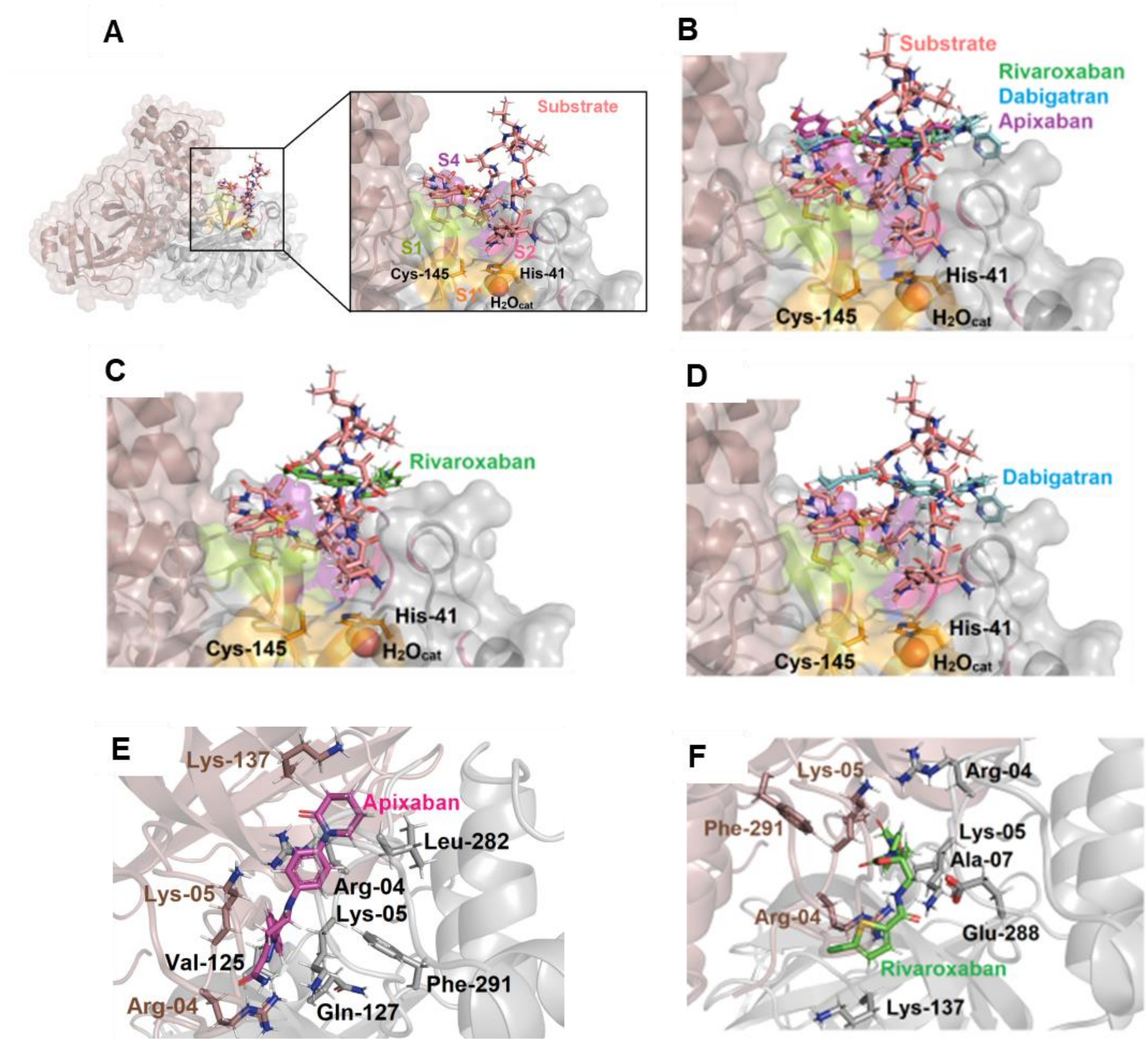

Supplementary Figure S2. Best docking pose (ChemPLP function) for the interaction between M^{pro} (**A**) modified peptide DABCYL-KTSAVLQSGFRKME-EDANS as substrate, (**B**) substrate-anticoagulants, (**C**) substrate-rivaroxaban, and (**D**) substrate-dabigatran into the active site of protease. Best docking pose (ChemPLP function) for the interaction between M^{pro} (**E**) apixaban in the allosteric binding site, and (**F**) rivaroxaban into the dimer interface of the protease. Substrate, rivaroxaban, dabigatran, and apixaban are in stick representation in beige, green, cyan, and pink, respectively, while the catalytic water (H_2O_{cat}) is in sphere. The monomers which constitute the active M^{pro} structure are in cartoon representation in brown and gray. Elements' color: hydrogen, nitrogen, oxygen, sulfur, and chloro are in white, dark blue, red, yellow, and dark green, respectively.

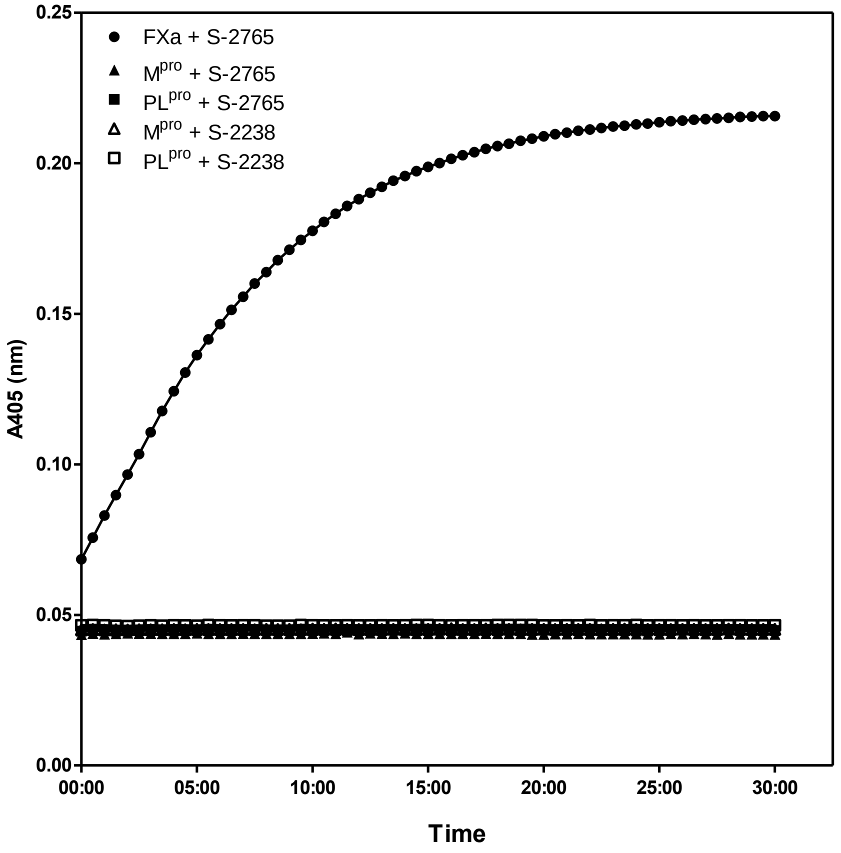

Supplementary Figure S3. Cleavage of chromogenic substrates S-2765 and S-2238 by M^{pro} and PL^{pro}. M^{pro} (10 nM) and PL^{pro} (10 nM) were incubated with S-2765 or S-2238 (0.2 mM final concentration) and the kinetic of the reactions was monitored by measuring the absorbance at 405 nm for thirty minutes at room temperature. (l) indicate control performed in the presence of FXa (1.25 nM). Results are expressed as mean values ±SEM of duplicates.

Supplementary Table

Supplementary Table S1. The 50% effective concentration (EC_{50} for MOI 0.1), 50% cytotoxic concentration (CC_{50}), and selectivity index (SI) of the anticoagulants and remdesivir in Calu-3 cells.

Drug	EC_{50} (μM)	CC_{50} (μM)	SI
**Remdesivir**	0.0305 ± 0.0031	512 ± 30	1.68×10^4
**Rivaroxaban**	5.90 ± 0.30	553 ± 28	93.7
**Dabigatran**	6.94 ± 0.35	756 ± 38	109
**Apixaban**	1.84 ± 0.09	491 ± 25	267
